# Raman Gas Sensor for Hydrogen Detection via Non-Dispersive and Dispersive Approaches

**DOI:** 10.3390/s25134190

**Published:** 2025-07-05

**Authors:** Fabio Melison, Lorenzo Cocola, Luca Poletto

**Affiliations:** National Research Council of Italy, Institute for Photonics and Nanotechnologies, CNR-IFN, Via Trasea 7, 35131 Padova, Italy; lorenzo.cocola@cnr.it (L.C.); luca.poletto@cnr.it (L.P.)

**Keywords:** Raman spectroscopy, hydrogen detection, gas sensing, optical sensor, field-deployable instrumentation, non-dispersive Raman

## Abstract

The current solicitude in hydrogen production and its utilization as a greenhouse-neutral energy vector pushed deep interest in developing new and reliable systems intended for its detection. Most sensors available on the market offer reliable performance; however, their limitations, such as restricted dynamic range, hysteresis, reliance on consumables, transducer–sample interaction, and sample dispersion into the environment, are not easily overcome. In this paper, a non-dispersive Raman effect-based system is presented and compared with its dispersive alternative. This approach intrinsically guarantees no sample dispersion or preparation, as no direct contact is required between the sample and the transducer. Moreover, the technique does not suffer from hysteresis and recovering time issues. The results, evaluated in terms of sample pressures and camera integration time, demonstrate promising signal-to-noise ratio (SNR) and limit of detection (LOD) values, indicating strong potential for direct field application.

## 1. Introduction

Hydrogen is currently taken into consideration as a renewable energy carrier. The motivations lie in the fact that it is light, storable, reactive, has a high energy content per unit of mass, and it can be readily produced at an industrial scale. Supplying hydrogen to industrial users is now a major business globally. Demand for hydrogen in its pure form is around 100 million tons per year (MtH2/yr) [1].

Moreover, the use of fossil fuels as the primary energy source is unsustainable due to the rapid depletion of natural reserves and the economic challenges associated with exploiting untapped resources. In addition, the growing attention to greenhouse gas emissions is directing the governments, and thus the industrial sector, towards employing renewable and greenhouse gasses free sources of energy [2,3,4].

Hydrogen is highly reactive; on Earth, it is always found combined with other elements. In particular, it is mainly extracted from water, hydrocarbons, and biomasses, and not all the processes used to produce hydrogen are greenhouse gas-free in the overall balance of input energy and output emissions. For example, hydrogen production through the oxidative conversion of hydrocarbons, a well-established industrial process, generates not only hydrogen, but also carbon monoxide and carbon dioxide. However, when this process is applied to the by-products of other industrial activities, it becomes possible to retrieve hydrogen from greenhouse gases that are more impactful than carbon oxides [5]. So, the needs in measuring the hydrogen content in gas mixtures cover its entire lifecycle.

From its production and towards its transportation and utilization, it is essential to accomplish the following: (i) Maintain compliance with regulatory requirements such as safety and quality standards. (ii) Optimize the efficiency of the apparatus involved in its conversion to energy, e.g., industrial applications and fuel cells require a proper level of purity. (iii) Guarantee safety in the environments involved in its transit, preventing and quantifying any losses in the process.

Different techniques are applied in hydrogen measurements depending on the particular requirements to be satisfied: sensitivity, response and recovery time, full-scale, cross-talk with impurities, etc. The primary methods adopted by the industrial sector include the following: (i) Electrochemical sensors: amperometric, potentiomentric, or conductoctrometric. Their transducer operations are based on the hydrogen oxidation on an electrode. These sensors are widely used and they are cost-effective, portable, and they can reach high accuracy (<5 ppm); on the other hand, their recovery time depends on the sensor temperature, they suffer from cross-sensitivity with other molecules such as carbon monoxide and methane, and they could undergo hysteresis over time [6,7,8,9]. (ii) Thermal conductivity detectors (TCDs) are generally used in gas chromatography to measure the content of a particular element or molecule. This type of transducers uses the inherently different thermal conductivity characteristic of gases to measure concentrations. They represent a non-specific, robust, and mature technology, commonly used as reference instrumentation. TCDs lead to accurate results with low LODs and wide dynamic ranges; conversely, due to their non-specificity, they require some sort of sample manipulation and in general, they are cost-impactive for some applications [10,11,12]. (iii) Catalytic combustion sensors are widely used in industrial safety monitoring to detect flammable gases, including hydrogen. Deep interest and research are growing in developing new and increasingly high-performing materials for use as a transductor. Their principle is based on the hydrogen oxidation on a catalytic surface, and the gas concentration is proportional to the temperature variation of the surface itself. This type of sensor has a limited dynamic range and requires direct contact between the sample and the sensor itself [12,13,14,15]. (iv) Tunable diode laser absorption spectroscopy (TDLAS) has been recently applied to hydrogen detection. TDLAS is based on the photon absorption by molecules at determined energy levels. The method is extensively employed to reliably measure the concentration of gasses such as water, carbon dioxide, methane, etc. It has been recently applied to hydrogen detection by exploiting its absorption lines at 2121.8 nm and 4712.9 nm. Some research studies demonstrated its applicability to hydrogen detection by providing detection limits which comply with the safety standards and regulations [16,17].

## 2. Materials and Methods

Raman spectroscopy uses light to probe vibrational (and rotational in case of gases) energy levels of molecules. Differently from IR spectroscopy, which is based on the absorption of photons at the precise energy levels of those of the vibrations, Raman spectroscopy is based on the inelastic scattering of the incident photons. The outcoming photons are shifted in energy (loss or gain) in relation to the vibrational fingerprints of the molecules hit by the radiation [18,19,20,21,22]. 

In gaseous samples analysis, Raman spectroscopy results is a non-invasive approach as it only requires a laser beam to pass through the sample and the optical windows necessary to collect the scattered light. As in the IR spectroscopy, the direct contact between the sample and the detecting section of the instrument can be easily avoided; this method does not require any direct interaction with the sample. It does not require sample preparation; only dust removal is necessary to prevent particle-induced scattering of the laser beam, which could compromise data acquisition. In addition, there are no issues related to time recovery, as once the sample exits from the laser–sample interaction volume, the system is immediately ready for the subsequent analysis. Ultimately, because the Raman emission of water does not spectrally overlap with that of hydrogen, removing water vapor is unnecessary. However, it remains essential to avoid thermal conditions that could lead to water condensation.

Thanks to recent advancements in CMOS industrial cameras (such as improved quantum efficiency, back-illuminated sensors, and reduced thermal noise), high-power laser technology, and the ability to operate in the visible range, Raman spectroscopy is gaining increased attention as a technique for analyzing gaseous samples. This technology is poised to play an expanded role in industrial settings, as prior studies demonstrated its applicability both within and beyond research laboratories [23,24,25,26,27,28].

In this work, a Raman system designed for the detection of hydrogen, nitrogen, and oxygen is presented. Previous studies demonstrated the feasibility of accurately detecting trace amounts of hydrogen by implementing a non-dispersive approach combined with a multi-pass cell to achieve optical powers close to 100 W. With integration times ranging from 10 min to 12 h, limits of detection (LODs) as low as 20 ppb have been achieved at a sample pressure of 2 bar [29]. Other studies have shown that it is possible to use commercial instrumentation to detect hydrogen, reaching LODs as low as 775 ppm, with acquisition times ranging from 1 to 15 min [30]. This work aims to highlight the feasibility of developing an instrument specifically designed for industrial use, featuring operating pressures and integration times compatible with typical process control dynamics. The system is conceived to be as compact and straightforward as possible, while still maintaining the performance level of a scientific-grade instrument. The system has been designed with future optimization and integration into industrial production in mind, thereby enabling potential field applications of Raman-based techniques.

The presented system is characterized by a non-dispersive approach as the Raman scatterings of the three molecules under analysis are selected by employing different band-pass filters, which allow the proper wavelengths to pass through the filters themselves and to be collected by a camera. The performances achievable with this design are compared to a dispersive approach which employs a diffraction grating spectrometer to analyze the acquired spectrum simultaneously.

In Raman spectroscopy, both dispersive and non-dispersive approaches exhibit inherent advantages and limitations related to their respective designs. These differences become particularly significant when considering the complexity of the required components and the alignment procedures during assembly, factors that are especially critical in the context of industrial replication of instrumentation.

Dispersive systems typically rely on a combination of objectives, diffraction gratings, entrance slits, detectors, and in some cases, mirrors, which collectively result in a more complex optical and mechanical design. The optimal alignment of all these components is crucial to ensure efficient signal collection and high spectral resolution. Achieving this often requires precise matching in the numerical apertures of the optical elements to minimize signal losses, which in turn frequently necessitates the use of custom, non-commercial components. Moreover, the assembly process is time-consuming and highly sensitive to mechanical tolerances. Although modern dispersive systems have become more compact and robust, Raman spectroscopy of gaseous samples often demands custom-designed spectrometers with high optical throughput and tailored configurations specific to the application. This leads to a high degree of system complexity, which poses significant challenges for large-scale manufacturing and field deployment.

On the other hand, non-dispersive systems, which commonly employ optical filters, are generally characterized by a reduced number of optical components. As a result, these systems feature a simplified structure that does not require diffraction gratings or entrance slits. The detector is typically positioned directly along the optical axis of the collecting optics, effectively reducing both system complexity and the likelihood of misalignment during operations. Consequently, the overall design is easier to implement, and the optical throughput can be readily enhanced using only commercially available components. These characteristics make the non-dispersive configuration particularly appealing for industrial applications, where repeatability, robustness, and ease of assembly are essential requirements. Non-dispersive systems are, however, limited in terms of spectral coverage, as they can only analyze the emissions selected by the optical filters. This makes them particularly well suited for applications in which only a few Raman peaks are of interest. In such cases, the trade-off in spectral range is outweighed by the practical benefits of reduced system complexity, improved manufacturability, and enhanced mechanical stability.

Both the acquisition systems here presented are designed to record and analyze the Raman Stokes features of the three molecular species. Given the simple two-atom structure of these molecules, their Raman emissions result in distinctive peak-shaped spectra. In the case of complex-shaped spectra, with overlapped emissions, the non-dispersive approach could be difficult to implement or even impossible without the use of several filters and cross-talk characterizations in the spectral regions sampled by the band-pass filters.

The Raman shifts of oxygen, nitrogen, and hydrogen are as follows: 1556 cm^−1^, 2331 cm^−1^, and 4161 cm^−1^, respectively. Hydrogen has also other rotational emission peaks, closer to the pump wavelength, not taken into consideration in this work [18,19,31,32].

Using a laser source with emissions centered at a wavelength of 532 nm, the resulting Raman peaks will be at wavelengths centered at about 580 nm, 607 nm, and 683 nm for oxygen, nitrogen, and hydrogen, respectively. The filters selected for this study are commercially available (Thorlabs, Newton, NJ, USA), hard-coated band-pass filters with central wavelengths of 580 nm, 610 nm, and 680 nm. All the filters are characterized by a FWHM of 10 nm, with transmittance peaks exceeding 90% of the incoming light. Additionally, their optical densities outside the transmission bandwidth are greater than 5.

**Figure 1:**
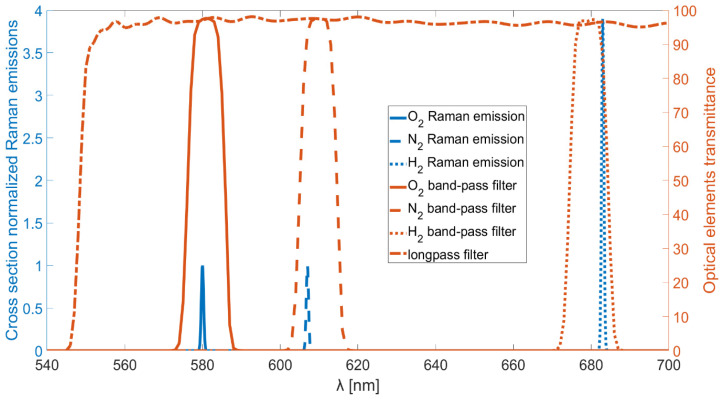
Raman emissions and filter transmittances.

An explanatory graph is reported in Figure 1, in which the relevant Raman emissions normalized to the proper cross sections are drawn in the figure with blue lines. The Raman emission not involved in this study, the rotational Raman emission of hydrogen, is not reported. The transmittances of the band-pass filters and the long-pass filter used in the non-dispersive setup are also reported with red lines. The vibrational Raman emissions of hydrogen and nitrogen are not perfectly centered with the transmission bands of their respective band-pass filters. However, the hydrogen emission peak corresponds to approximately 87% transmittance of its filter, while the nitrogen emission peak corresponds to about 93% transmittance of its filter.

Possible interfering molecules in hydrogen production environments include methane, carbon monoxide, and carbon dioxide: (i) Methane is characterized by a strong Raman emission (cross-section ≈ 8.6) corresponding to the ν_1_ symmetric C–H stretching mode, located around 2917–2919 cm^−1^. With the excitation source used in this setup, the corresponding Raman-shifted wavelength is 629.7 nm, well outside the transmission bands of the filters implemented. However, methane is a more complex molecule than nitrogen, oxygen, or hydrogen, which leads to additional emissions, such as the ν_2_ bending mode at 1535 cm^−1^, corresponding to 579.3 nm. This emission nearly overlaps with the Raman peak of oxygen. Therefore, for applications requiring simultaneous detection of methane and oxygen, an additional filter centered at 630 nm should be employed to collect the methane ν_1_ signal, and a cross-sensitivity analysis must be performed prior to deployment. Similarly, methane also exhibits weak Raman features in the 3500–4000 cm^−1^ range, which is attributed to overtones, hot bands, and combination bands. These weak features may interfere with the hydrogen signal, and an analogous cross-sensitivity evaluation, as described for oxygen, is required. (ii) Carbon monoxide has a Raman peak at 2143 cm^−1^ (cross-section ≈ 0.9), corresponding to 600.5 nm. This peak does not interfere with any of the molecular signals considered in this study. (iii) Carbon dioxide exhibits a Fermi doublet with peaks at 1388 cm^−1^ (ν_1_) and 1285 cm^−1^ (2ν_2_), and with Raman cross-sections of approximately 1.1 and 0.8, respectively. The corresponding wavelengths are 574.4 nm and 571.0 nm. The first peak lies within the ~8% transmission range of the oxygen filter, while the second falls entirely outside the transmission bands of all the used filters. In the presence of CO_2_, to ensure accurate measurement, it is advisable to introduce an additional filter centered at 570 nm and to perform a O_2_-CO_2_ cross-sensitivity calibration prior to operation. These observations hold true as long as commercially available filters with an FWHM ≈ 10 nm are used. For specific applications requiring a higher degree of selectivity, it remains possible to employ custom-made filters specifically designed to achieve perfect alignment between the spectral peaks and passbands while simultaneously minimizing their FWHM as much as possible.

On the other side, the dispersive system acquires the spectrally dispersed Raman emissions. To distinguish overlapping spectral components, such as those above discussed, it is necessary to acquire a dataset of pure calibrations to be used in a fitting procedure. Cross-sensitivity is inherently mitigated by the fitting algorithm, which determines the optimal combination of calibration spectra that best matches the acquired spectrum [26,28].

**Figure 2:**
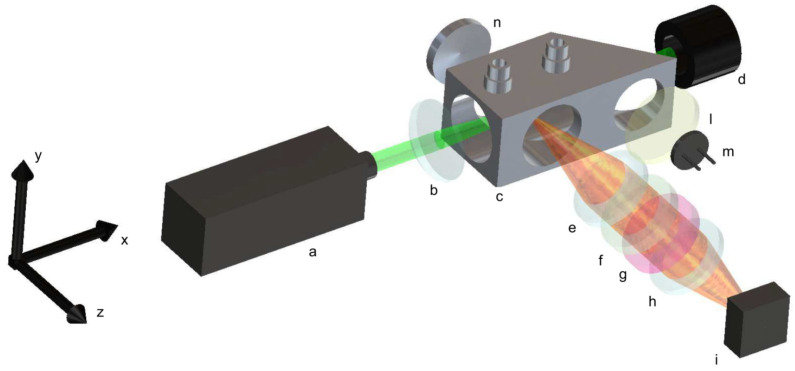
System schematic.

A simplified schematic of the non-dispersive system is reported in Figure 2. The system excites spontaneous Raman emission via a 532 nm DPSS doubled Nd:YAG continuous wave laser (a); such a source provides an optical power of about 1.5 W, and its emission linewidth is less than 0.1 nm, with a spatial mode near to TEM00. The collimated radiation emitted by the laser source is characterized by a linear polarization, with the electric field oriented in parallel to the *y*-axis shown in Figure 2. The beam is focused into the gas cell, (c) thanks to a 1-inch diameter, 50 mm focal length, anti-reflection coated focusing lens (b). The gas cell is custom-designed, and it is realized in brass. It incorporates the housing to accommodate the focusing lens directly into its body. All optical windows present in the gas cell are of a 1-inch diameter, broadband anti-reflection-coated, 3 mm-thick, and mounted in the gas cell body; two windows are placed in correspondence to the beam focus; on the output side of the cell, an optical window is placed to form a 45° angle with the incoming beam. After this window, the beam is terminated into a beam dump (d). The partial reflection is transmitted through an additional window and collected by a photodiode (m) used to normalize eventual drifts in the optical power of the laser source during acquisitions. A light diffuser (l) is placed before the photodiode to homogenize the collected light.

The collecting optics is composed by two, 1-inch-diameter Hastings achromatic triplets (e, h) with focal lengths of 40 mm, leading to an effective aperture f/1.66. A long-pass filter (f) with a 550 nm cut-off wavelength and a band-pass filter (g), whose transmittance is centered at the wavelength of the Raman emission to be detected, is placed between the two achromatic triplets, where the light is collimated. To acquire different Raman emissions, the band-pass filters are mounted on a filters wheel station. The distances between the gas cell center, the two achromatic triplets, and the CMOS sensor of the camera are adjusted to recreate an image of the laser beam focused in the gas cell center onto the camera sensor. The camera (i) is an industrial un-cooled CMOS 2.3 MP with a pixel area of 5.86 × 5.86 µm^2^, which is based on the SONY IMX249 monochromatic sensor. An aluminum spherical mirror (n), with a focal length of 25 mm, is placed on the opposite side of the collecting optics at a distance of 50 mm. With this mirror, the emission scattered towards the opposite side of the camera is refocused in the gas cell center and then redirected towards the collecting optics. Its presence contributes by almost doubling, except for losses and non-idealities, the signal recorded by the camera sensor.

The dispersive setup used for the comparison is characterized by the same coupling optics composed by the two Hastings triplets (e, h). The coupling optics recreate an image of a laser beam focused in the gas cell onto the focal plane of a slit-less diffraction grating spectrometer, which provides about 11 nm/mm spectral dispersion onto the same camera used in the non-dispersive setup. The spectrometer consists of two fixed focal length objectives and a diffraction grating, adopting the same optical design successfully tested for Raman emission from natural gas [28]. The entrance objective is a 50 mm f/2, while the output objective is a 25 mm f/1.4; they are both directed towards the diffraction grating to form an angle of 71°. The diffraction grating has a groove density of 1200 grooves/mm and a blaze wavelength of 750 nm (Edmund 43-210). The long-pass filter (f) is positioned in the input arm of the spectrometer, between the input objective and the diffraction grating, where the light entering through the slit is collimated; the slit is adjusted to minimize the stray light coming from the gas cell, while avoiding vignetting of the laser beam image projected into it (1.2 mm along the y axis). The effective input focal ratio of the spectrometer is f/2.8, limited by the aperture of the output objective. In terms of light collected by the camera, there is about 40% in signal losses if compared to the non-dispersive setup. The spectral range covered by the CMOS camera allows the simultaneous acquisition of the Raman emission of oxygen, nitrogen, and hydrogen, although a clear vignetting due to the output objective is observed on the lines closed to the edges of the CMOS camera. To avoid vignetting, hydrogen and nitrogen-oxygen have been acquired separately in consecutive acquisitions by tilting the grating in order to have the Raman lines be acquired in the central region of the CMOS camera.

The dimensions of the non-dispersive setup are 35 cm × 10 cm × 25 cm along the x, y, and z axis, respectively. While the dimensions of the dispersive setup are 35 cm x 15 cm x 30 cm along the same axis. These volumes could be slightly reduced by re-design and optimizations. Furthermore, these dimensions are compatible with the manufacturing of portable instrumentation. In a practical case, the instrument could be housed in a frame, for safety and reliability reasons, and transported into a suitcase.

## 3. Experimental Structure

To analyze the system’s performance, a certified gas mixture from a bottle has been pressurized into the gas cell volume at three different pressures. Consequently, the molecular Raman emissions have been acquired with the two setups and the signals have been elaborated as discussed, subsequently. The certified gas bottle (RISAM GAS) composition is as follows: hydrogen 2% *v*/*v*, nitrogen 77.1% *v*/*v*, and oxygen 20.9% *v*/*v*. No mixture manipulation has been performed, only dust removal via a porous filter (5 µm).

The tests have been performed at different absolute pressures and integration times in the ranges of 1–3 bar and 2–10 s. For each condition, 100 camera frames have been acquired to determine system performance. In addition, 100 frames have been acquired for each camera integration time with the gas cell pumped down to 2 mbar by a membrane pump for the determination of the background levels. Furthermore, each image is corrected by the corresponding camera dark frame and finally normalized to its photodiode reading to take into account possible variations in the laser intensity.

The mixture into the gas cell is injected via a needle valve. A digital absolute manometer (Druck DPI 104, General Electric, Boston, MA, USA) measures the effective cell pressure.

**Figure 3:**
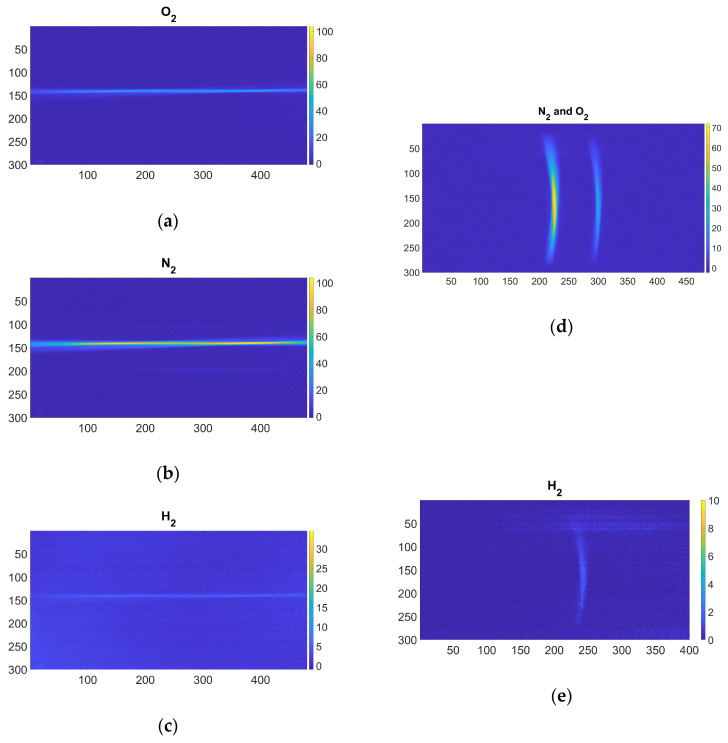
Image of Raman emission inside the cell using O_2_ filter (**a**), N_2_ filter (**b**), and H_2_ filter (**c**). (**d**) Spectrum of N_2_ (left emission) and O_2_ (right emission) emissions. (**e**) Spectrum of H_2_ emission.

Figure 3 shows examples of (4 × 4 binned) frames acquired with both systems. In the dispersive system, the emission is diffracted by the grating, and it is also possible to appreciate the Petzval field curvature [33].

The standard deviations of the residual signal in images acquired with the gas cell pumped to 2 mbar were considered as the system’s background noise in the absence of gas molecules. The presence of a specific molecule is detected when its signal exceeds at least three times this value.

## 4. Discussion

**Figure 4:**
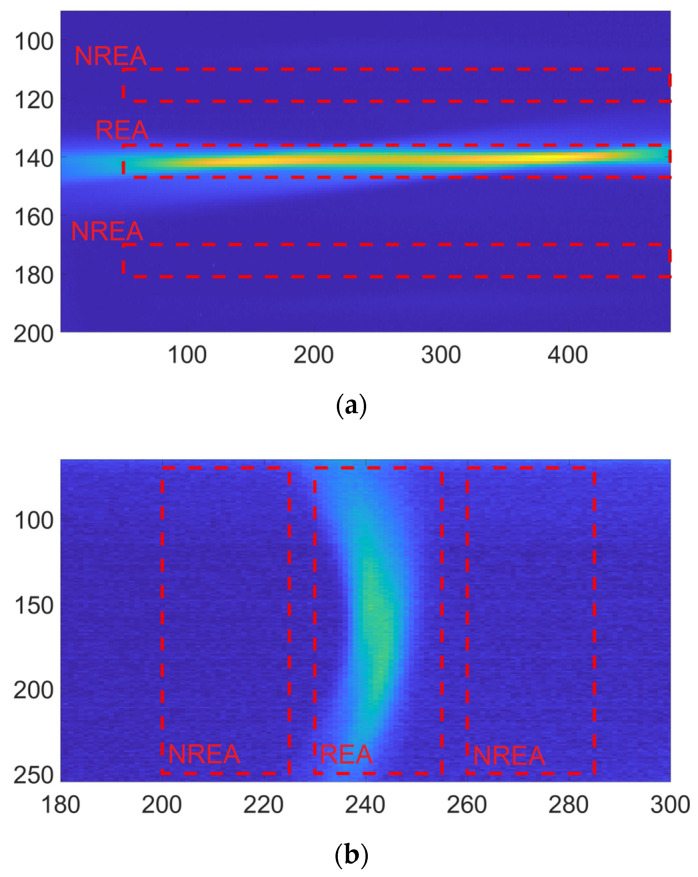
(**a**) Image of the Raman emission inside the gas cell using the H2 filter. (**b**) Spectrum of the H2 emission. In both figures, REA and NREAs are identified by the dashed rectangles.

As discussed in Section 3, after the average dark frame subtraction, the signal is defined as the average of the pixel values (PV) in the frame portion in which the Raman emission is collected, minus the average value in the regions, with the same area, in which there is not detected emission. Expression (1) resumes the data elaboration for a single frame of the camera after the average dark frame subtraction:

(1) $$Signal=(\sum_{REA}PV-\sum_{NREA}PV)/A,$$

where REA is the Raman emission area, NREA is a non-Raman emission area close to REA with the same area A. In Figure 4, the hydrogen Raman emissions and the related REA and NREA are reported for both the setups. To define the REA and NREA regions for each setup, frames were averaged along the laser propagation direction. This yields a one-dimensional array whose length corresponds to the number of sensor columns in the non-dispersive case and to the number of sensor rows in the dispersive case. The resulting signal is then approximated with a Gaussian fit. The start and end points of the REA regions are identified as the mean ± 3σ of the fitted function. This procedure was applied to the most intense signals for each molecule (10-s exposure time and highest pressure); in this way, even lower-intensity signals are fully included. Otherwise, part of the signal might be excluded from the analysis. The NREA regions were identified adjacent to the REA regions, where no signal or stray light are detected on the sensor, and are defined to have the same area as the corresponding REA regions.

The same elaboration has been applied to the frames acquired with the vacuum pump connected to the gas cell and subsequently used for the LODs calculations.

**Figure 5:**
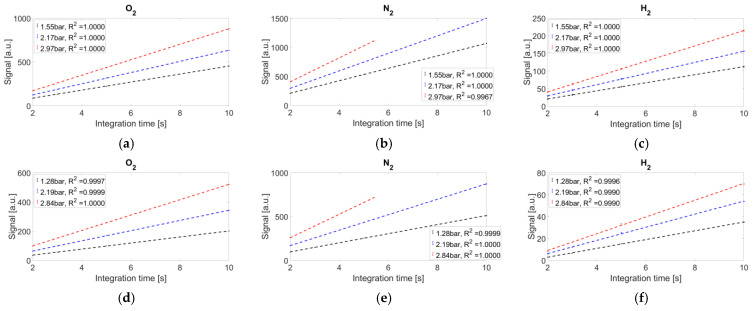
Average recorded signals, standard deviations, and linear interpolations. (**a**–**c**) Non-dispersive system and (**d**–**f**) dispersive system. The dashed lines represent the linear interpolation of each dataset.

The average recorded signals, with their error bars (standard deviations), are reported in Figure 5.

The coefficients of determination are reported in the legend of each graph. Under the experimental conditions of 10 s camera integration time and 3 bar gas cell pressure, the nitrogen signals are saturated in both setups. Nevertheless, both systems exhibit high linearity.

The measurement uncertainties, defined as expressed in Equation (2), are listed in Table 1 and Table 2.

(2) $$err=conc[\%]\left(\frac{std(Signals)}{mean(Signals)}\right),$$

The signals of the non-dispersive setup are higher than the dispersive one by a factor ≈1.8 for O_2_, ≈1.7 for N_2_, and ≈3.1 for H_2_. At the same time, their uncertainties are higher, which is due to the fact that the stray light is increasing. Accurate inspection of the frames reveals that they exhibit a significant amount of stray light if compared to those acquired with the dispersive system under the same experimental conditions. Indeed, the presence of the entrance slit and the further grating dispersion contribute to mitigating the stray light compared to the non-dispersive setup.

**Figure 6:**
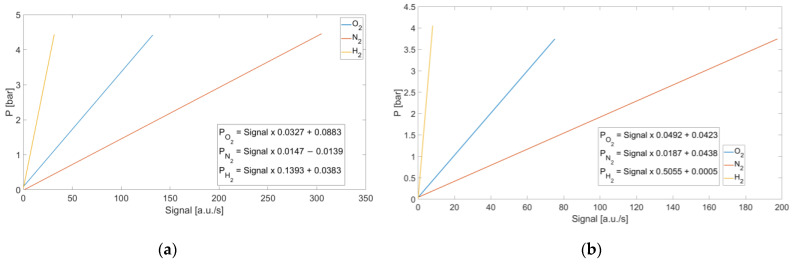
Calibration curves of signal normalized to the integration time vs. molecular pressure. (**a**) Non-dispersive system. (**b**) Dispersive system. The signal is expressed in units of counts per second, calculated by dividing the number of counts obtained through the aforementioned processing by the integration time of the camera expressed in seconds.

To characterize the mixture pressures as a function of the recorded signal, calibration curves were calculated for both systems. For each acquired pressure, the corresponding signals were normalized with respect to the integration time. This approach allows the calibration curves to be defined independently of the integration time, which is a known parameter that can be set via software. Consequently, the pressure of the mixture can be determined for any selected integration time. The calibration curves are shown in Figure 6. The *x*-axis represents the signal normalized to the integration time, while the *y*-axis indicates the corresponding mixture’s pressure values.

To determine the LODs of the two systems, the background noise must be quantified. In this study, it is calculated as three times the standard deviation of the net signal acquired under vacuum conditions. The minimum detectable signal is defined as three times the noise standard deviation. In the following, we concentrate on the hydrogen LOD normalized to 1 bar; to do so, the signals acquired at the lowest pressures are normalized to 1.55 bar and 1.28 bar for the non-dispersive and dispersive systems, respectively. Normalized signal to 3σ background noise ratios (SBNR_3σ_) are reported in Table 3.

The LOD is expressed by Equation (3):

(3) $$LOD=\frac{conc\%}{{SBNR}_{3\sigma }}=\frac{conc\%}{\frac{{Signal}_{1bar}}{3*BackgroundNoise}}$$

Table 4 resumes the LODs for both the non-dispersive and dispersive approaches.

The LODs of the non-dispersive system result are lower than the dispersive setup. This is due to the lower number of optical elements, leading to higher efficiency and to the increased luminosity.

It is worth mentioning here that the LOD is inversely proportional to the sample pressure. Therefore, for high-pressure applications, the LODs reported in Table 4 can be substantially reduced.

Results here presented may stimulate the use of such systems as practical applications in the fields of hydrogen safety and fuel cells. The lower flammability limit (LFL) for hydrogen is 4 vol% in air and 10 vol% in nitrogen. Many safety applications require LOD as low as 1% of the LFL, corresponding to 400 ppm. When referring to the LFL in air, relevant applications include trucks, indoor fueling (area monitoring), residential settings, production facilities (area monitoring and in-unit), road vehicles, battery backup systems (in buildings), general battery backup, outdoor storage, and indoor storage [34,35].

## 5. Conclusions

Two Raman effect optical setups for hydrogen detection have been presented in this paper. Both the systems do not require consumables, sample handling (except filter for dust removal), or preparation. No oxygen or water removal are required as their Raman emission does not affect the hydrogen signal. By adjusting the camera integration time, both the systems provide full-scale measurements, as they are able to measure the entire dynamic range from their LODs up to the 100% concentration, avoiding saturations. The method has no recovery time and hysteresis as once the sample is flushed out from the gas cell, it is immediately ready to analyze a new sample without any memory. The non-dispersive approach leads to lower LODs in a simpler and more compact optical setup.

The presented system could still take advantage of some technological improvements, such as multi-pass cell configuration, operation at shorter laser pump wavelengths, and the adoption of a cooled camera in order to increase SNR, thus further lowering the LODs. Future improvements of the system will be aimed at enhancing measurement repeatability and lowering the LODs.

## Figures and Tables

**Table 1:** Non-dispersive system uncertainties.

	O_2_ (20.9%)			N_2_ (77.1%)			H_2_ (2.0%)		
	1.55 Bar	2.17 Bar	2.97 Bar	1.55 Bar	2.17 Bar	2.97 Bar	1.55 Bar	2.17 Bar	2.97 Bar
2 s	0.111	0.092	0.066	0.202	0.194	0.099	0.110	0.070	0.062
3 s	0.105	0.058	0.061	0.179	0.142	0.098	0.074	0.058	0.041
5 s	0.068	0.053	0.041	0.150	0.110	0.121	0.044	0.033	0.023
10 s	0.049	0.043	0.035	0.168	0.094		0.021	0.015	0.011

**Table 2:** Dispersive system uncertainties.

	O_2_ (20.9%)			N_2_ (77.1%)			H_2_ (2.0%)		
	1.28 Bar	2.19 Bar	2.84 Bar	1.28 Bar	2.19 Bar	2.84 Bar	1.28 Bar	2.19 Bar	2.84 Bar
2 s	0.049	0.040	0.031	0.097	0.104	0.101	0.041	0.024	0.020
3 s	0.036	0.039	0.025	0.083	0.121	0.077	0.028	0.016	0.012
5 s	0.030	0.018	0.021	0.099	0.059	0.071	0.015	0.009	0.007
10 s	0.028	0.031	0.025	0.092	0.110		0.010	0.005	0.005

**Table 3:** H_2_ SBNR_3σ_, ratios between signals and background noise referred to 1 bar sample pressure.

	Non-Dispersive: H_2_ SBNR	Dispersive: H_2_ SBNR
2 s	38	16
3 s	48	24
5 s	80	40
10 s	161	66

**Table 4:** H_2_ LODs expressed in ppm, values referred to 1 bar sample pressure.

	Non-Dispersive: H_2_ LOD	Dispersive: H_2_ LOD
2 s	530	1240
3 s	420	830
5 s	250	505
10 s	125	305

## Data Availability

The raw data supporting the conclusions of this article will be made available by the authors on request.

## Abbreviations

The following abbreviations are used in this manuscript:

SNR	signal-to-noise ratio
LOD	limit of detection
TCDs	thermal conductivity detectors
TDLAS	tunable diode laser absorption spectroscopy
FWHM	full width half maximum
REA	Raman emission area
NREA	non-Raman emission area
SBNR	signal-to-background-noise ratio
